# Regulation of the *scp* Genes in the Cyanobacterium *Synechocystis* sp. PCC 6803—What is New?

**DOI:** 10.3390/molecules200814621

**Published:** 2015-08-12

**Authors:** Otilia Cheregi, Christiane Funk

**Affiliations:** Department of Chemistry, Umeå University, Umeå SE-90187, Sweden; E-Mail: christiane.funk@chem.umu.se

**Keywords:** *Synechocystis* 6803, SCP, high light regulatory motif (HLR1), NtcA, HIP1

## Abstract

In the cyanobacterium *Synechocystis* sp*.* PCC 6803 there are five genes encoding small CAB-like (SCP) proteins, which have been shown to be up-regulated under stress. Analyses of the promoter sequences of the *scp* genes revealed the existence of an NtcA binding motif in two *scp* genes, *scpB* and *scpE*. Binding of NtcA, the key transcriptional regulator during nitrogen stress, to the promoter regions was shown by electrophoretic mobility shift assay. The metabolite 2-oxoglutarate did not increase the affinity of NtcA for binding to the promoters of *scpB and scpE*. A second motif, the HIP1 palindrome 5ʹ GGCGATCGCC 3ʹ, was detected in the upstream regions of *scpB* and *scpC.* The transcription factor encoded by *sll1130* has been suggested to recognize this motif to regulate heat-responsive genes. Our data suggest that HIP1 is not a regulatory element within the *scp* genes. Further, the presence of the high light regulatory (HLR1) motif was confirmed in *scpB*-*E*, in accordance to their induced transcriptions in cells exposed to high light. The HLR1 motif was newly discovered in eight additional genes.

## 1. Introduction

Cyanobacteria are photosynthetic prokaryotes found in almost every terrestrial or aquatic habitat: they occur in oceans, as well as in fresh water, on rocks, in the deserts or soil and even as endosymbionts of lichens living in Arctic ice. Cyanobacteria have a high adaptive potential to different stresses; modifications of their gene expressions are the first responses to perturbations in the environment and set the plan for physiological strategies.

In the cyanobacterium *Synechocystis* sp. PCC 6803 (hereafter referred to as *Synechocystis* 6803) genes encoding the small CAB-like proteins (SCPs, also called high light induced proteins or Hlips) have been shown to be up-regulated during exposure to a variety of stress conditions. The SCP protein family contains five members and includes proteins of low molecular weight (~6–8 kDa), called ScpB-ScpE (HliC/Ssl1633, HliA/Ssl2542, HliB/Ssr2595, HliD/Ssr1789), as well as ScpA, the C-terminal extension of the ferrochelatase enzyme HemH (Slr0839) [1]. Additionally, LilA (Slr1544) is considered a member of the SCP family [2], even though it is related to a lesser extent. With their high homology to the light harvesting antenna complexes of higher plants, the membrane-inserted SCPs belong to the light-harvesting-like proteins. SCPs contain the CAB domain, which is important for chlorophyll-binding of their plant relatives, and ScpB-E have been shown to be able to bind pigments* in vitro* [3], ScpE even* in vivo* [4]. The exact function of the SCPs is not known, however, a series of scientific reports has shown the SCP family members to associate with Photosystem II (PSII) [4,5,6,7,8], where they might stabilize nascent PSII proteins [9]. Recently two SCP proteins have been shown to be part of a novel high-light-inducible carotenoid binding protein complex (HLCC), which protects thylakoid membranes against extensive photooxidative damage [10]. Their role in regulating the tetrapyrrole biosynthesis is acknowledged [6,11,12,13]. SCPs also might prevent the formation of singlet oxygen in damaged PSII reaction centers [14]. Performing integrative analysis of the *Synechocystis* 6803 transcriptome, the *scp* genes were grouped in a single expression cluster [15,16], therefore their expression pattern is similar beyond their individual regulation under different stress conditions.

In this report we analyze the upstream regions of the *scp* genes and describe two new regulatory elements: an NtcA binding motif in the promoter regions of *scpB* and *scpE*, and a palindrome motif in the promoter regions of *scpB* and *scpC*. NtcA functions as master regulator of nitrogen metabolism in both, nitrogen fixing and non-fixing cyanobacteria [17]. In the absence of ammonia, the preferred cyanobacterial nitrogen source, NtcA activates genes required for the use of alternative nitrogen sources, such as nitrate or N_2_. The regulatory role of NtcA extends beyond nitrogen acquisition, it is important in the general regulation of nitrogen carbon or iron metabolism [18], and for heterocyst differentiation of N_2_-fixing bacteria [19]. Recent data point to an even greater importance of NtcA as global regulator, in an overexpressing mutant rewiring of the primary metabolism has been observed [20]. The C-terminal DNA-binding domain of the NtcA protein recognizes the promotor signature motif GTA-N8-TAC, localized 20–24 bp upstream of the -10 box TA-N3-T. The metabolite 2-oxo-glutarate has been found to stimulate NtcA binding [21,22]. Although 2-oxoglutarate levels are directly related to the balance of carbon and nitrogen metabolism, only in *Anabaena* sp. PCC 7120 and *Synechococcus elongatus* PCC 7942 they have been shown to allosterically control NtcA binding to DNA [23].

The palindrome 5′ GCGATCGC 3′, known as Highly Iterated Palindrome (HIP1), has been initially discovered in *Synechococcus* PCC 6301 within the *smtB* gene, where it exists four times inside the protein coding region and three times outside of it [24]. Not only in the *smtB* gene, but also in other sequenced genes available in databases at that time the HIP1 palindrome was detected and proposed to promote gene rearrangements that would confer selective advantage in changing environments [24]. Computational analysis established HIP1 to be polyphyletic, occurring in distinct cyanobacterial lineages [25]. In some prokaryotic genomes, a statistically significant difference of HIP1 copies was observed in the coding compared to the non-coding regions [26], still, a functional role of HIP1 in the coding regions was excluded. A recent study by Elhai [27] related the high occurrence of the HIP1 motif to the presence of methyl transferases, and suggested DNA methylation to happen on HIP1 sites. An EMSA assay to identify proteins binding to the HIP1 motif in *Synechococcus* PCC 7942 was unsuccessful [25], however, recently Krishna and co-workers [28] identified in *Synechocystis* 6803 a transcription factor encoded by *sll1130* that regulates *slr1788* by binding to the HIP1 motif located in the upstream region of this gene. 

A High Light Regulatory 1 (HLR1) element [29] common to the promoter regions of high-light inducible genes *psbA2*, *psbA3*, *nblA*, *hliA* in *Synechocystis* 6803, has been identified in all *scp* genes by relating the expression of *scp* genes with their promoter profile [30]. The protein binding to the HLR1 motif of *scpD* was identified as RpaB [31]. RpaB was also found to interact with promoters of genes encoding proteins of Photosystem I during low light and to stimulate their expression [32,33]. We have analyzed the promoter regions of the *scp* genes with the aim to identify elements important for their regulation. Here we describe two newly discovered regulatory motifs, the NtcA motif and the HIP1 palindrome within *scp* gene promoters and upstream regions. Using EMSA and DNA-pull down assays we have verified the functionality of these motifs. We also newly identified eight genes that have the already characterized HLR1 motif in their upstream regions.

## 2. Results and Discussion

### 2.1. NtcA Binds to the Promoter Regions of scpB and scpE

**Figure 1:**
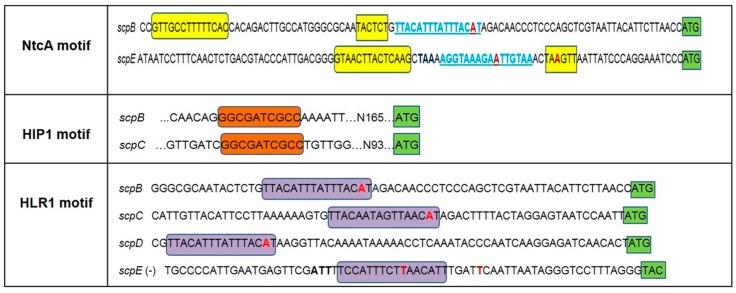
Summary of motifs present in the upstream regions of the *scp* genes of *Synechocystis* 6803. The location and sequence of the NctA motif is highlighted by **yellow** boxes, the HIP1 palindrome by **orange** boxes and the HLR1 motif is highlighted by **purple** boxes. The NtcA motif of *scpE* gene is shown at the positive strand of the promoter region. In light blue and underlined is the location of HLR1 motif indicated additionally to the NtcA motif. The HLR1 motif within the *scpE* promoter region is shown at the negative strand for a better visualization of the motif sequence. Nucleotides shown in **red** bold represent the starting point of transcription as described in the literature (two putative starting points of transcription were detected for *scpE*) while the translation start codon is highlighted by **green** boxes; nucleotides shown in black bold indicate the stop codon of *slr1090*, the gene upstream of *scpE*.

SCPs are known to be involved in the acclimation response to the majority of stresses experienced by cyanobacteria—excess light, nutrient depletion and temperature changes. In an attempt to fully understand their role, modifications of their gene expression and the effect of regulatory elements have to be taken into account. To learn more about their expression pattern and the possible functions of the corresponding SCP proteins, the promoter and upstream regions of the *scp* genes were analyzed using *scp* gene clustering information from the CyanoEXpress and Synergy web servers and the MEME toolkit to predict DNA motifs. Analysis of the DNA sequences upstream of the *scp* genes revealed the presence of a previously undetected motif; the promoters of *scpB* and *scpE* contain a putative NtcA binding site (Figure 1). Within the *scpB* promoter the NtcA motif is situated upstream of a HLR1 motif, its second domain being separated from the HLR1 motif only by one nucleotide (Figure 1). In the *scpE* promoter, the two domains of the NtcA motif surround the HLR1 motif. Further on, the TAN_3_T module of the NtcA motif, necessary for the binding of RNA polymerase, is located at the -10 element. NtcA therefore might act as a repressor of gene transcription, as it has been observed for the *gifA* and *gifB* genes of *Synechocystis* 6803 [34]. 

**Figure 2:**
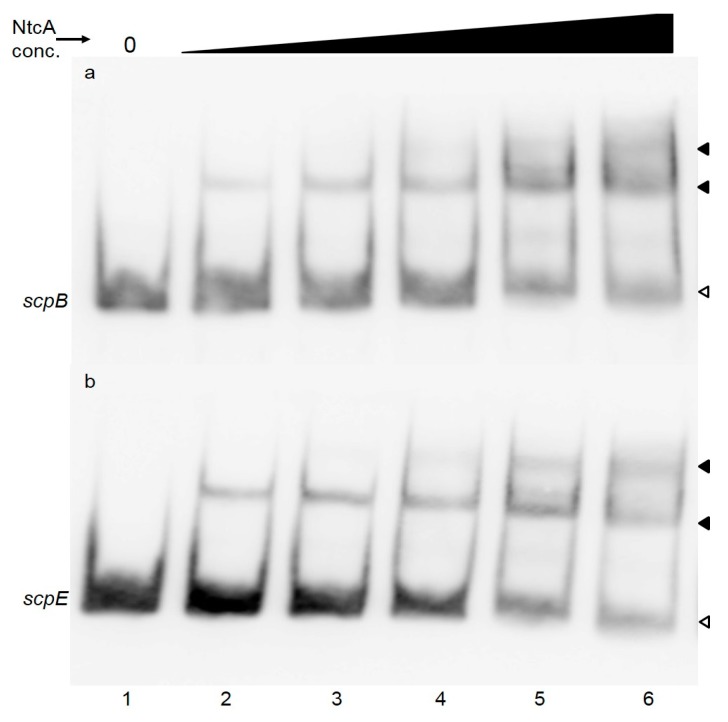
EMSA assay to verify NtcA binding to the *scp*B (**a**) or *scp*E (**b**) promotor regions. Purified recombinant NtcA from *Synechocystis* 6803 (2.5, 5, 7.5, 15 or 30 pmol—lanes 2–6) were incubated with 2 ng of labelled PCR fragments of the promoter regions of *scp*B (110 bp) (**a**) or *scpE* (90 bp) (**b**). In lane 1 labeled PCR product was loaded without any addition. White arrowheads point to unbound labeled DNA, black arrowheads to NtcA-DNA complexes.

To verify whether NtcA indeed binds to the newly identified promoter sites we performed electrophoretic mobility shift assays with recombinant NtcA from *Synechocystis* 6803. DNA fragments of the promoter regions of *scp*B and scpE with a size of approximately 100 bp were amplified by PCR, labeled with digoxigenin (DIG), and then incubated in the presence or absence of the NtcA protein. The complexes were then separated on native polyacrylamide gels. Gel-shift assays demonstrating the interaction of NtcA protein with *scp*B or *scp*E promoters are presented in Figure 2a,b. To verify the specificity of the interaction between NtcA and the *scp* promoter regions, the binding reaction was additionally performed with a DNA fragment sharing no similarity to the NtcA binding motif (the control DIG-labeled 39mer oligonucleotide provided in the kit), in the presence of a specific competitor (unlabeled probe that had been added in excessive amount) (Supplementary Figure S1A). Despite using a partially purified NtcA protein (Supplementary Figure S2A) no shift was detected when the non-specific competitor was used, an indication that the contaminating proteins do not have DNA-binding activity. The specificity of the interaction was further tested using a DNA fragment of 110 bp of the coding region of *scpB* (Supplementary Figure S1B). 

Presence of NtcA binding sites in the promoter regions of *scpB* and *scpE* has not been reported earlier and we are able to confirm binding of NtcA to these motifs. The NtcA motifs are located in close proximity to the core promoter sites within both genes, similar to the NtcA binding sites found in the promoters of *gifA* and *gif B* of *Synechocystis* 6803 [34], *rbcL* of *Anabaena* PCC 7120 [35] and *myc* genes of *Microcystis* PCC 7806 [36]. Transcription of these genes is repressed by NtcA. The expression of *scpB* and *scpE* under nitrogen depletion correlates with the expression of *ntcA* gene: while *ntcA* is gradually repressed during the 6, 12 and 24 h of nitrogen depletion (0.02, −0.25, −0.2, respectively), the inhibition of *scp*s is gradually released (−3.34, −1.96, −0.96) [37]. Nitrogen stress seems to induce both *scp* genes [37,38,39] and their respective proteins [40]. It is worth noting that only the promoters of *scpB* and *scpE* contain the NtcA binding motif, but the expression of *scpC* and *scpD* was reported to be affected as well by nitrogen stress [37]. NtcA binding sites of these two *scp* genes might have escaped our detection, or more likely, transcription of some *scp* genes might affect the transcription of others, as has been noted earlier [2]. In *Anabaena* sp. PCC 7120 the largest direct regulon has been identified for NtcA, binding to 2424 sites in 2153 genes [41]. *Anabaena* sp. PCC 7120 contains 10 *scp* genes, and only one of these has an NtcA binding site [41]. *Prochlorococcus* strains MED4 and MIT 9313 contain 22, respectively 9 *scp* genes [42], but only three (and respectively, two) genes have been shown to be significantly up-regulated under nitrogen stress and only one (and respectively, two) have NtcA binding sites in their promoters [42]. Despite the accumulating evidence for NtcA regulation of certain *scp* genes ([41,42] and present report) the specific role of the respective proteins during nitrogen stress is not known. However, there are a number of reports that allow to relate the NtcA motif in the *scpB* and *scpE* genes with pigment biosynthesis: ScpB and ScpE proteins have been shown to affect tetrapyrrole biosynthesis at a level prior to 5-aminolevulinic acid (ALA) biosynthesis [9,11,12], maybe at the level of glutamyl-tRNA (glu-tRNA) [11]. Glutamyl-tRNA is a substrate for protein synthesis and also the first substrate for the production of 5-aminolevulinic acid, the universal precursor for the synthesis of porphyrins. α-Ketoglutarate (2 oxoglutarate) represents the carbon skeleton for nitrogen fixation that is converted by the GS/GOGAT cycle into glutamate, the precursor of glutamyl-tRNA. In *Synechococcus* sp. PCC 7942, NtcA finely regulates the Glu-tRNA expression by binding to one of the three NtcA binding sites present in the upstream region of this gene. It is thus reasonable to hypothesize that as global regulator, NtcA might finely control more than one step of pigment biosynthesis, possibly through ScpB and ScpE. 

Alternatively, to allow response to general stresses, a subset of the *scp* genes might have evolved as NtcA targets and in this way ensure rapid up-regulation during nitrogen stress [42]. They were also shown to be involved in photo-protection and stabilization of the photosystems [4].

### 2.2. 2-Oxoglutarate does not Stimulate NtcA Binding

**Figure 3:**
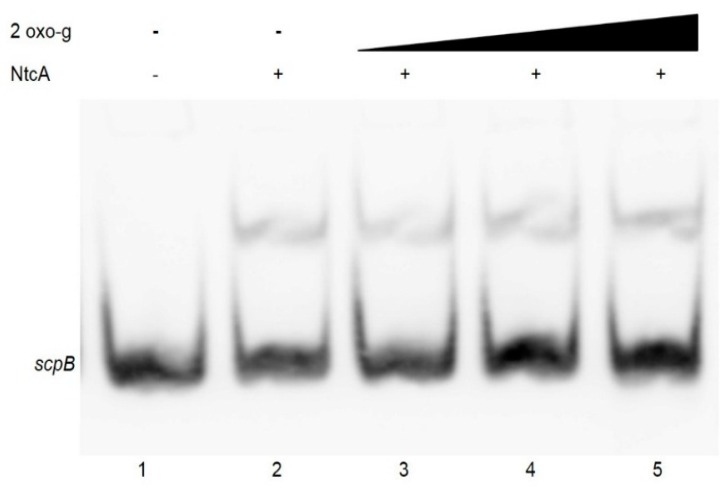
Effect of 2-oxoglutarate on NtcA binding to the *scpB* promoter sequence. EMSA was performed in the presence of 2 ng of a 110 bp labelled PCR fragment of the *scp*B promoter region*,* 7.5 pmol purified recombinant NtcA, 5 mM MgCl_2_ and 2-oxoglutarate at concentrations of 0.2, 0.6 or 1 mM (lanes 3, 4, 5). In lane 1 labeled PCR product of the *scpB* promoter region was loaded without any addition.

In nitrogen-fixing cyanobacteria the metabolite 2-oxoglutarate has been shown to function as a positive effector for binding of NtcA to DNA fragments [22]. To investigate the effect of 2-oxoglutarate on NtcA binding to the *scp*B and *scp*E promoters of *Synechocystis* 6803, the binding reaction was supplemented with 0.2–1 mM 2-oxoglutarate in the presence of 5 mM MgCl_2_, in accordance to the previous reports [22]. Presence of 2-oxoglutarate in the binding assay, however, did neither stimulate NtcA binding affinity to the *scpB* (Figure 3), nor to the *scpE* promoter (not shown); no change in the intensity of the labeled probe no additional band shifts were observed either (Figure 3).

Presence of 2-oxoglutarate and/or the PipX mediated connection to PII have been found to be important for NtcA binding to the promoter regions in nitrogen-fixing cyanobacteria [23,43] and in *Synechocystis* 6803 the level of 2-oxoglutarate is maximal during nitrogen starvation [44]. Using Surface Plasmon Resonance (SPR) also in *Synechocystis* 6803, the presence of 2-oxoglutarate was shown to positively affect NtcA binding. In this study we were unable to detect enhanced NtcA binding to the promoters of *scpB* and* scpE*, even in the presence of 2-oxoglutarate at high concentrations. SPR is far more sensitive than EMSA and enhanced binding might have been below our detection limits, however, it is also possible that in *Synechocystis* 6803 the stimulation of 2-oxoglutarate for NtcA binding is weaker than in nitrogen-fixing cyanobacteria. Recent structural studies on the NtcA-2-oxoglutarate complex revealed that 2-oxoglutarate is not essential for the DNA-binding capacity of NtcA, but affects its binding strength [23,43]. Alternatively, additional factors, such as PipX protein, might be necessary for binding [43].

### 2.3. scpB and scpC Contain a HIP1 Motif

A second motif found in the upstream regions of the *scp* genes is a palindrome consisting of eight nucleotides described in the literature as Highly Iterative Palindrome (HIP1, Figure 1). The function of this motif, which occurs with high frequency in the genome of *Synechocystis* 6803, is unknown [25,45]. HIP1 was identified in the upstream regions of *scp*B and *scp*C as an extended palindrome of 10 nucleotides. In these two genes it is located roughly 100 base pairs upstream of the annotated start codon.

**Figure 4:**
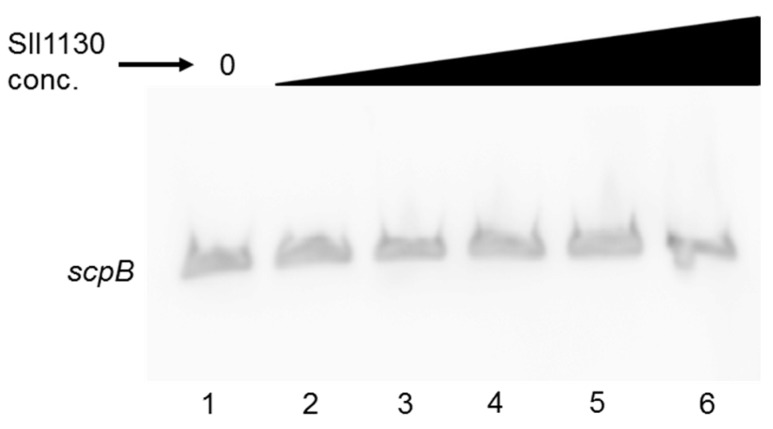
EMSA assay to test the binding of *Sll1130* to the HIP1 motif within the upstream region of *scp*B. Increasing amounts (0, 5, 10, 15, 30 or 100 pmol; lanes 1–6) of purified recombinant Sll1130 from *Synechocystis* 6803 were incubated with 2 ng of a 110 bp labelled PCR fragment of *scp*B.

Recently in *Synechocystis* 6803 the transcription factor Sll1130 was shown to repress expression of *slr1788* by binding to its HIP1 motif [28]. As the upstream regions of *scp*B and *scp*C also contain such HIP1 motif, binding of Sll1130 to these regions was investigated using EMSA. As shown in Figure 4, purified His-tagged Sll1130 protein failed to bind to the 156 bp PCR fragment of the *scpB* upstream region. Adding increasing amounts of the protein did not affect the binding.

**Figure 5:**
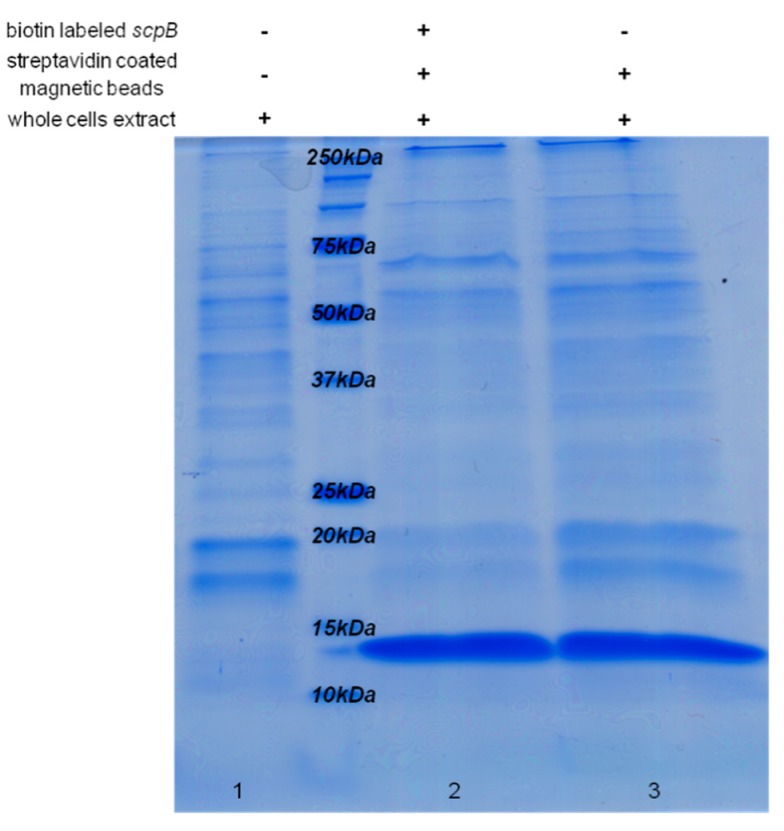
Coomassie-stained SDS-PAGE after DNA pull-down assay, to analyze the functionality of the HIP1 upstream binding site. 150 μg of *Synechocystis* 6803 whole cell extract was incubated with 250 μg magnetic beads and 1.2 μg biotin-labeled PCR fragment (156 bp) of the upstream region of *scpB* (lane 2, “+++”), or without labeled PCR probe (lane 3, “−++”), and then separated by SDS PAGE. 2 µg of total *Synechocystis* 6803 cell protein extract (lane 1, “−−+”) was loaded as control. The intense band with molecular weight of 11 kDa (lanes 2, 3) corresponds to streptavidin covering the magnetic beads used in the assay.

The functionality of the HIP1 binding site was then investigated by a DNA pull-down assay [46], which should detect any proteins/transcription factors binding to the HIP1 motif of the *scp*B upstream region. A 156 bp biotin-labeled fragment of the upstream region of *scpB* was synthesized by PCR and bound to streptavidin-coated magnetic Dynabeads^®^ M-280 (Invitrogen Dynal AS, Oslo, Norway). After 15 min incubation with *Synechocystis* 6803 whole cell extracts, the beads were washed to eliminate unspecific bindings. The proteins were released by heating up the beads up to 95 °C and analyzed by SDS-PAGE (Figure 5). No difference could be observed in the protein pattern obtained in the presence or absence of labeled DNA, therefore binding of any protein to the biotin-labeled DNA fragments was not detected. However, one cannot exclude the situation in which a protein would bind the DNA fragment but due to the very low amount would not be detected by Coomassie staining. The intense protein band with molecular weight of 11 kDa (Figure 5, lanes 2 and 3) corresponds to streptavidin. An additional band with molecular weight of around 70 kDa was observed in the non-labeled sample, corresponding to bovine serum albumin (BSA) used in the binding buffer to suppress non-specific interactions.

In *Synechocystis* 6803 the Sll1130 protein has been described as a novel transcription factor that negatively regulates the expression of heat-responsive genes by recognizing the GGCGATCGCC palindrome within their upstream regions [28]. The ubiquitous palindrome, named HIP1 [24,45] was correlated to the presence of DNA methyltransferases and a function within DNA methylation [27]. We calculated the HIP1 motif to occur in the genome of *Synechocystis* 6803 at least 1028 times in upstream regions, but also 2695 times in coding regions of genes. Without taking into account that the first value might be underestimated due to inaccuracies in operon annotations, about one third of all genes in the *Synechocystis* 6803 genome contain the HIP1 motif in their upstream regions. It is difficult to imagine that such an arbitrary distributed motif [25,26,45] would be recognized in some genes by a specific transcription factor, as shown by Krishna* et al.*, [28]. Instead of binding to the HIP1 motif, Sll1130 might bind to another motif present in the 283 bp upstream fragment used in the EMSA assay by these authors [28]. The data presented in this study suggest that the HIP1 motif is non-functional in the upstream regions of *scpB* and *scpC*: in our set-up neither Sll1130 nor other transcription factors bind to it. Therefore our data are in agreement with the data described by Robinson* et al.*, [25] performing EMSA with the HIP1 motif within *Synechococcus* sp. PCC 7942.

### 2.4. HLR1 Motif

Figure 1 further shows the presence of the regulatory element HLR1 in the promoter regions of the *scp* genes [30]. It is interesting to note that the HLR1 motif is located very closely upstream of the transcription start of the genes, at a distance of only 3–13 bp. Besides the *scp*s, we were able to identify eight other genes with this HLR1 motif in their promoter regions that previously have escaped detection (Table 1).

**Table 1:** Promoter sequences of *Synechocystis* 6803 with newly identified HLR1 motifs using the MEME web server.

Gene ID	Gene Product	HLR1	*p*-Value	Position Relative to the Translational Start Site	Strand
ssl2542 *	ScpB	TTACAATAGTTAACAT	1.16 × 10^−8^	−42	+
*sll1483*	Hypothetical protein	TTCACAAAAATTTATA	1.41 × 10^−8^	−63	+
*sll0157*	Hypothetical protein	TTACAATCGTTTACAA	1.54 × 10^−8^	−50	+
*sll2012*	SigD	TGAGAAAACTTTACAA	6.38 × 10^−8^	−44	−
*slr1894*	MrgA	TAACAAAATTTAACAC	1.97 × 10^−8^	−32	−
*slr0320*	Hypothetical protein	TTAAGATATATTACAA	6.57 × 10^−8^	−78	+
*slr1687*	Hypothetical protein	TAACAAAACTTTATAA	1.07 × 10^−8^	−47	−
*ssl3044*	Probable ferredoxin	TAACAAAACTTTATAA	1.07 × 10^−8^	−142	+
*ssl2162*	Unknown protein	TGTAAACCTTTGTTAA	1.13 × 10^−8^	−75	−

* Identified by [30] and included in the table for sequence comparison. “+” sense and “−“antisense strands.

The HLR1 motif has been shown to be involved in either transcriptional repression or stimulation, depending on its location in the target promoters. In high light inducible genes, like the *scp* genes, the HLR1 binding motif is located within the core promoter region [29] (Figure 1) leading to down-regulation of gene transcription under normal light conditions. RpaB, the cognate transcription factor, binds to the HLR1 motif under standard and low-light conditions and prevents the interaction between RNA-polymerase and the core promoter. The current model for regulation through RpaB is that, during increasing light intensities, Hik33 mediates dephosphorylation of RpaB-P, which promotes dissociation of RpaB from HLR1 motif and results in derepression of negatively controlled genes [47,48]. In RpaB positively regulated genes, such as PSI genes, RpaB binds to the HLR1 motif and interacts with RNA polymerase to enhance genes transcription under low-light [32]. In these genes HLR1 motif is located upstream of the core promoter region. The promoter architecture of *psaA/B* genes is more complicated than of other PSI genes, having three HLR1 motifs in their upstream regions that affect two high light responsive promoters. The regulatory role of HLR1 motif is not necessarily determined by its location; base substitutions in HLR1 sequence affect local structural properties and spatial conformation with different effects on P1 and P2 promoters of *psaA*/*B* genes [49].

Various microarray experiments (reviewed by [50]) studying the transcription of *scp* genes during different light intensities revealed their transcription to be positively correlated to the strength of irradiance. However, also the concentration of CO_2_ exposure of the cells varied in these experiments ranging from air level (0.04%) to 3% CO_2_. It has been shown that cells grown in the presence of high CO_2_ have a lower PSI/PSII ratio compared to air-grown cells [51]. Cells grown at high CO_2_ concentrations therefore mimic cells exposed to short (up to 6 h) high light treatment [52]. Cells exposed to 500 μmol photons m^−2^·s^−1^ and 3% CO_2_ adapt to both, high light and high CO_2,_ by increasing transcription of *scpB*, *scpD* and *lilA* genes. In fact, transcription of these three genes was most up-regulated within the whole microarray experiment [53]. During decreasing CO_2_ concentrations the *scp* genes were down-regulated (at 60 and 180 min) [54]. In the same conditions, two of the eight genes containing the newly described HLR1 motif (Table 1, *sll0157* and *slr0320*) were shown to have a similar expression pattern as the *scp* genes, suggesting that dissociation of RpaB from the HLR1 motif allowed their transcription during CO_2_ depletion. Most of these newly identified genes (Table 1) encode proteins functioning in the general stress response, their up-regulated expression was observed during high light [55], salt stress [56], peroxide stress [57], acid stress [58] and CO_2_ limitation [54]. Detection of the HLR1 motif in the hypothetical and unknown proteins mentioned in Table 1 will facilitate investigations on their functions and identification of the regulatory network they belong to. Sll1483 is a periplasmic protein similar to the transforming growth factor induced protein with hypothetical function. Even though *sll1483* is up-regulated at almost all stress conditions investigated in *Synechocystis* 6803, the function of the corresponding protein still is unknown. Proteins encoded by s*ll1483* and *sll2012*, as well as the SCPs have been suggested to be specifically regulated during salt and hyperosmotic stress by Hik33 through RpaA (Rre31) [59]. During low temperature stress [60] or high light [61] this set of genes is regulated by Hik33 through RpaB (Rre26) (reviewed in [62]); very likely the newly discovered HLR1 motif in their promoters is, therefore, relevant for their regulation through RpaB.

## 3. Experimental Section

### 3.1. Strains and Growth Conditions

*Synechocystis* 6803 wild type strain was grown at 30 °C in BG-11 medium at a light intensity of 30 μmol photons m^−2^·s^−1^. The cells were cultured in vented flasks, with agitation, at ambient CO_2_ level.

### 3.2. Protein Expression and Purification

The NtcA gene of *Synechocystis* 6803 was amplified from genomic DNA using the forward primer 5ʹ-AACGTCCATGGATCAGTCCCTA-3ʹ and reverse primer 5ʹ-CATAGAGGTACCT TAGGTAAACTG-3ʹ. The PCR product was inserted into the restriction sites *NcoI* and *ACC65I* of the plasmid petHIS_1a resulting in a coding region for an NtcA protein with N-terminal His_6_-tag.

*Escherichia coli* Rosetta cells carrying plasmid petHIS_NtcA were grown overnight in LB medium supplemented with kanamycin. Ten ml of this culture were used to inoculate 1 liter LB media containing 50 μg/mL kanamycin and grown at 37 °C with shaking. When the culture reached OD_600_-0.5, IPTG was added to a final concentration of 0.5 mM and cells were further incubated in the same conditions for another 2 h. The cells were harvested by centrifugation and resuspended in Ni-IMAC start buffer containing 20 mM Tris, pH 8.0, 0.5 M NaCl and 90 mM imidazol. The cells were disrupted by five cycles of sonication, 30 s each, centrifuged and the supernatant was loaded on a HisGraviTrap column equilibrated with buffer B containing 20 mM imidazole. Then the column was washed with 5 mL buffer B containing 40 mM imidazole. The bound proteins were eluted in 1 mL eluting fractions with buffer B containing 150 mM imidazole. The second elution fraction contained most of NtcA protein with highest purity. The *sll1130* gene was PCR-amplified using the primers described in the literature [28]. The amplified ORF of *sll1130* was inserted into pET MBP_1c at the NcoI and HindIII sites. The C-terminally His-tagged Sll1130 protein was expressed and purified under the same conditions as described above.

### 3.3. Electrophoretic Mobility Shift Assay (EMSA)

DNA fragments used in the electrophoretic mobility shift assay were obtained by PCR amplification (primers listed in Table 2) from genomic DNA. Primers scpB-Fw and scpB-R were used to amplify a fragment from the promoter region of the *scpB* gene with a size of 110 bp, and primers scpE-Fw and scpE-R were used to amplify a fragment of 90 bp from the promoter region of the *scpE* gene. A 156-bp DNA fragment from the promoter region of *scpB* containing the HIP1 site was amplified by PCR using the Fw-HIP1 and R-HIP1 primers.

**Table 2:** Primer sequences.

NtcA-Fw	5ʹ-AACGTCCATGGATCAGTCCCTA-3ʹ
NtcA-R	5ʹ-CATAGAGGTACCTTAGGTAAACTG-3ʹ
Sll1130-Fw	5ʹ-GCGCCATGGATACAATTTACGA ACAATTTG-3ʹ
Sll1130-R	5ʹ-GCGAAGCTTACCGAGTTTAAAAACATGGGG-3′
*scpB*-Fw	5ʹ-TTTGAGCCTAACATTATCCTCC-3ʹ
*scpB*-R	5ʹ-ATGTAATTACGAGCTGGGAGG-3ʹ
*scpE*-Fw	5ʹ-ACTCTGACGTACCCATTGAC-3ʹ
*scpE*-R	5ʹ-CTCATGGGATTTCCTGGGATA-3ʹ
*scpB*-CR-Fw	5ʹ-TAAATTTGGATTCACTGCTTTCG-3ʹ
*scpB*-CR-R	5ʹ-AGTGAAGTACACCTTGCCCAGAG-3ʹ
Fw-HIP1	5ʹ-ATCCTGGGACTGGAGAATCA-3ʹ
R-HIP1	5ʹ-CGGGTGGTCATAATTGGATTA-3ʹ

The DNA fragments were 3ʹ end labeled with DIG-11-ddUTP using the DIG Gel Shift kit from Roche (Roche Diagnostics GmbH, Mannheim, Germany). The DNA-binding reactions with NtcA or Sll1130 protein, were carried out according to the instructions of the manufacturer. Typically, 2 ng of labeled DNA was used in each binding reaction together with 1–7.5 pmol of purified recombinant proteins. When indicated, 2-oxoglutarate was added to the reaction mix. After incubation for 15 min at room temperature, the mixture was subjected to electrophoresis on a native 8% polyacrylamide gel. In competition assays, the same purified PCR product, but unlabeled, was used as competitor. The non-specific competitor was an unlabeled DNA fragment of 40 bp, provided with the DIG Gel Shift kit from Roche Diagnostics GmbH. The gels were blotted onto nylon membranes and processed according to the vendor’s instructions (DIG Gel Shift kit, Roche). Thereafter the labeled bands were visualized at room temperature using a LAS-3000 Luminescent Image Analyzer (Fujifilm, Tokyo, Japan).

### 3.4. Bioinformatic Analysis

DNA regions corresponding to 400 bp upstream of the start codon of the *scp* genes and other genes with similar expression pattern (as grouped by the CyanoEXpress and Synergy web servers) [15,16] were downloaded from Cyanobase [63,64] *Synechocystis* and submitted to the MEME program in search of new regulatory motifs. The program was run with the default set-ups. The program correctly identified the HLR1 motif in *scp* genes and other genes as well as the HIP1 palindrome. However, since the MEME program does not allow the detection of gapped motifs, the NtcA motif was overlooked and only observed after personal inspection of the same DNA regions. 

### 3.5. DNA Pull down Assay

To perform the DNA pull down assay streptavidin coated magnetic beads (Dynabeads^®^ M-280 Streptavidin) were used following the instructions of the manufacturer (Invitrogen Dynal AS, Oslo, Norway). The 156-bp DNA fragment from the upstream region of *scpB* (see description of the electrophoretic mobility shift assay) was amplified with the Fw-HIP1 primer being biotin-labeled. The immobilization of the biotin-labeled DNA fragment to the streptavidin magnetic beads was carried out according to the manufacturer’s instructions. Isolation of whole cell extracts was performed as described elsewhere [65]. Incubation of the magnetic beads in whole cell extract was performed as in [46] and [66]. The protein-DNA incubation buffer included: 250 μg beads bound to the biotin-labeled DNA fragment, 1 mg/mL poly (dI-dC), 0.6 mg/mL herring sperm DNA, 1% BSA, 5 mM MgCl_2_ and 150 μg whole cells extract proteins. Beads were washed three times in buffer C (20 mM Tris pH 8, 1 mM EDTA, 10% glycerol, 1 mM DTT, 50 mM NaCl) and resuspended in SDS sample buffer and heated at 95 °C for 5 min. Samples were analyzed by SDS-PAGE and stained with Coomassie blue.

## 4. Conclusions

This work focuses on the regulatory elements present in the promoter and upstream sequences of the *scp* genes that are affecting their transcription. We were able to (i) newly identify putative NtcA binding sites in the promoter regions of *scpB* and *scpE* genes and, using EMSA, we could show that NtcA indeed binds to these DNA regions; (ii) we have detected the HIP1 motif in the regions upstream of *scpB* and *scpC*, however, neither Sll1130, the transcription factor binding to this motif in *slr1788* [28] nor other transcription factors were found to bind to the HLR1 motif of *scpB* or *scpC.* We therefore suggest this element to be non-functional; (iii) we confirmed the presence of an HLR1 motif in the promoter sequence of four *scp* genes and additionally detected for the first time eight more genes containing this motif.

## Supplementary Materials

Supplementary materials can be accessed at: http://www.mdpi.com/1420-3049/20/08/14621/s1.
