# Supplementary material for: Regulation of the scp Genes in the Cyanobacterium Synechocystis sp. PCC 6803—What is New?
Source: Molecules. 2015 Aug 12;20(8):14621–37. doi: 10.3390/molecules200814621 (PMC6331805; doi:10.3390/molecules200814621)
Supplement: Supplementary file 1 [file molecules-20-14621-s001.pdf]

Supplementary Materials

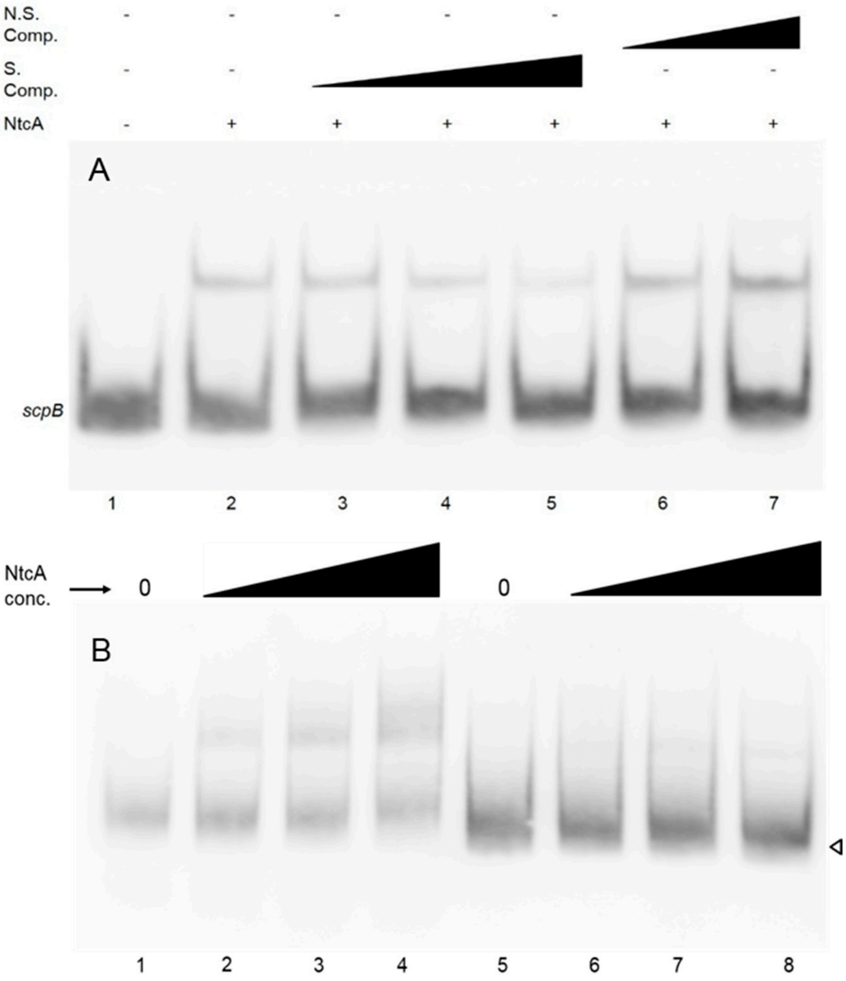

Figure S1. (**A**) Competitive assays in the absence (lane 1) or presence (lanes 2–7) of NtcA (5 pmol). Ten- (lane 3), 50- (lane 4) and 100-fold (lane 5) excess of unlabeled specific (S. comp., lanes 3–5) or 50- (lane 6) and 100-fold (lane 7) excess unlabeled non-specific (N.S. comp., lanes 6 and 7) competitor DNA was added to the reactions. The shift assay was performed with 2 ng of labeled *scpB* without any other addition. (**B**) Specificity assay. EMSA was performed with recombinant NtcA from *Synechocystis* 6803 and a 110 bp fragment of the promoter region (lanes 1–4) or a 110 bp fragment of the coding region of *scpB* (*scpB*-CR) (lanes 5–8). The shift assay was performed with 2 ng of labeled *scpB* or labeled *scpB*-CR and increasing amounts of NtcA: 2.5 pmol (lanes 2 and 6), 5 pmol (lanes 3 and 7) and 7.5 pmol (lane 4 and 8). White arrowhead indicates the free labeled DNA.

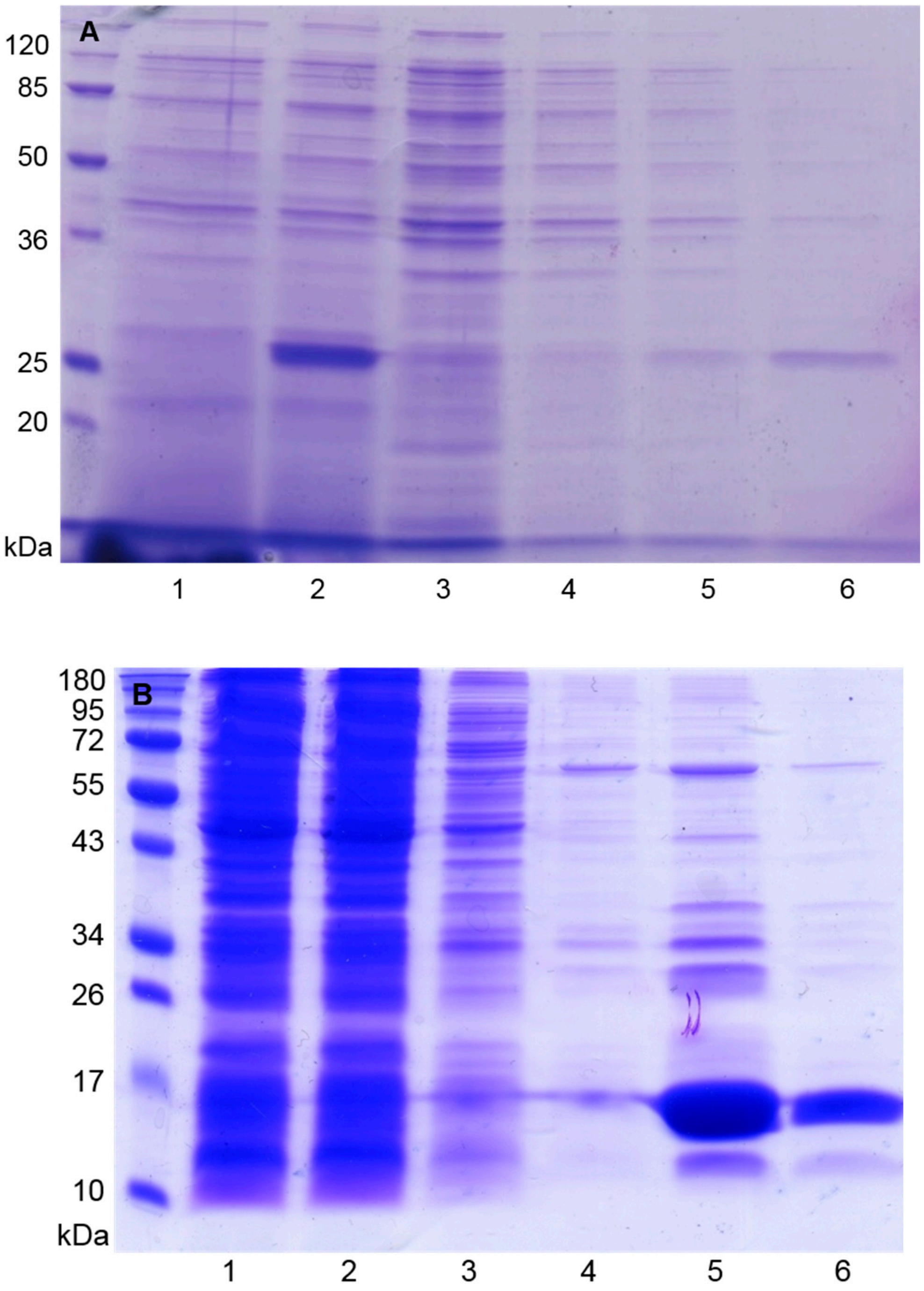

Figure S2. Purification of His-tagged NtcA (**A**) (25.8 kDa) using HisGraviTrap column. Samples before and after induction of protein expression (lanes 1 and 2), flow (lane 3), wash and elution (lanes 4–6) were separated on 15% SDS-PAGE and stained with Coomassie. Purification of His-tagged Sll1130 (**B**) (13.75 kDa) using HisGraviTrap column. Samples before and after induction of protein expression (lanes 1 and 2), flow (lane 3), wash and elution (lanes 4–6) were separated on 15% SDS-PAGE and stained with Coomassie.
